# Quantitative proteomics for identifying biomarkers for Rabies

**DOI:** 10.1186/1559-0275-10-3

**Published:** 2013-03-22

**Authors:** Abhilash K Venugopal, S Sameer Kumar Ghantasala, Lakshmi Dhevi N Selvan, Anita Mahadevan, Santosh Renuse, Praveen Kumar, Harsh Pawar, Nandini A Sahasrabhuddhe, Mooriyath S Suja, Yarappa L Ramachandra, Thottethodi S Keshava Prasad, Shampur N Madhusudhana, Harsha HC, Raghothama Chaerkady, Parthasarathy Satishchandra, Akhilesh Pandey, Susarla K Shankar

**Affiliations:** 1 Institute of Bioinformatics, International Technology Park, Bangalore, 560066, India; 2 Department of Biotechnology, Kuvempu University, Shimoga, 577451, India; 3 McKusick-Nathans Institute of Genetic Medicine, Johns Hopkins University, 733 N. Broadway, BRB 527, Baltimore, MD, 21205, USA; 4 Department of Biological Chemistry, Johns Hopkins University School of Medicine, Baltimore, MD, 21205, USA; 5 Amrita School of Biotechnology, Amrita Vishwa Vidyapeetham, Kollam, 690525, India; 6 Department of Neuropathology, National Institute of Mental Health and Neuro Sciences, Bangalore, 560029, India; 7 Rajiv Gandhi University of Health Sciences, Bangalore, 560041, India; 8 Manipal University, Madhav Nagar, Manipal, Karnataka, 576104, India; 9 Bioinformatics Centre, School of Life Sciences, Pondicherry University, Pondicherry, 605014, India; 10 Department of Neurovirology, National Institute of Mental Health and Neuro Sciences, Bangalore, 560029, India; 11 Department of Neurology, National Institute of Mental Health and Neuro Sciences, Bangalore, 560029, India; 12 Pathology, Johns Hopkins University School of Medicine, Baltimore, MD, 21205, USA; 13 Oncology, Johns Hopkins University School of Medicine, Baltimore, MD, 21205, USA

**Keywords:** Liquid chromatography, Mass spectrometry, Spectrum Mill, Hierarchical cluster, Post Exposure Vaccination

## Abstract

**Introduction:**

Rabies is a fatal acute viral disease of the central nervous system, which is a serious public health problem in Asian and African countries. Based on the clinical presentation, rabies can be classified into encephalitic (furious) or paralytic (numb) rabies. Early diagnosis of this disease is particularly important as rabies is invariably fatal if adequate post exposure prophylaxis is not administered immediately following the bite.

**Methods:**

In this study, we carried out a quantitative proteomic analysis of the human brain tissue from cases of encephalitic and paralytic rabies along with normal human brain tissues using an 8-plex isobaric tags for relative and absolute quantification (iTRAQ) strategy.

**Results and conclusion:**

We identified 402 proteins, of which a number of proteins were differentially expressed between encephalitic and paralytic rabies, including several novel proteins. The differentially expressed molecules included karyopherin alpha 4 (KPNA4), which was overexpressed only in paralytic rabies, calcium calmodulin dependent kinase 2 alpha (CAMK2A), which was upregulated in paralytic rabies group and glutamate ammonia ligase (GLUL), which was overexpressed in paralytic as well as encephalitic rabies. We validated two of the upregulated molecules, GLUL and CAMK2A, by dot blot assays and further validated CAMK2A by immunohistochemistry. These molecules need to be further investigated in body fluids such as cerebrospinal fluid in a larger cohort of rabies cases to determine their potential use as antemortem diagnostic biomarkers in rabies. This is the first study to systematically profile clinical subtypes of human rabies using an iTRAQ quantitative proteomics approach.

## Introduction

Rabies remains a significant public health challenge worldwide with 70,000 cases reported each year with a mortality rate of 100% in the absence of post-exposure prophylaxis [1]. The clinical presentation of rabies encephalitis in humans is in two distinct forms- the classical form which represents ~80% of all cases, also known as the “encephalitic or hydrophobic rabies” and the non-classical form which is also known as “paralytic” or “numb” rabies. The encephalitic form is characterized by the dramatic symptom of hydrophobia, which is a painful, violent, involuntary contraction of the diaphragmatic, accessory respiratory, pharyngeal and laryngeal muscles initiated by swallowing of liquids. In the paralytic form of rabies, the characteristic feature is flaccid paralysis and weakness, which often results in misdiagnosis with diseases such as Landry/Guillain Barre Syndrome [2-4]. Moreover, paralytic symptoms can also be observed in post-vaccinal encephalitis that occurs in some cases following administration of Semple vaccine as post-exposure prophylaxis [5,6]. In both the encephalitic and paralytic rabies cases, the duration of survival after the onset of symptoms is rarely greater than 7 days [4]. Until recently, Semple vaccination was the only mode of vaccination in countries such as India, where rabies is relatively common [7,8]. The onset of encephalomyelitis that occurs in some patients as a post-vaccination complication is due to an inflammatory response in the host to brain tissues contained in the Semple vaccine [9,10].

Clinical diagnosis of rabies is a challenge in the absence of specific laboratory diagnosis, and confirmation of diagnosis is often following postmortem examination of brain. Negri bodies are cytoplasmic inclusion bodies within rabies infected neurons and histopathological techniques such as Sellers staining [11] are used to demonstrate Negri bodies. However, only 50% of the brain samples show Negri bodies on histological evaluation compared to the fluorescent antibody test for rabies viral antigen, which is positive in almost 100% cases. The specificity of Negri bodies has also been challenged owing to the detection of inclusion bodies indistinguishable from Negri bodies in non-rabid tissues. Because of the low specificity and sensitivity, World Health organization (*WHO*) no longer recommends demonstration of Negri bodies for confirming a diagnosis of rabies [12]. Other diagnostic tests include fluorescent antibody test [13], rabies tissue culture infection test (RTCIT) [14] and the mouse inoculation test (MIT) [15], which are used for detecting viral particles. Although the fluorescent antibody test is widely used, it requires use of brain tissue and hence can only be performed postmortem. Some of the other available methods such as RTCIT and MIT are time consuming and less practical from a therapeutic perspective [12]. Thus, more sensitive methods of antemortem diagnosis of this fatal disease is essential.

There are two major sources for discovery of protein biomarkers - body fluids such as cerebrospinal fluid (CSF) and serum or the primary site of disease, which is the brain in cases of neurodegenerative diseases and neuroinfections. Brain tissue remains a limiting factor as the brain specimens are not obviously not accessible for antemortem diagnosis. Although not applicable for antemortem biomarker testing, the post-mortem brain tissue is of use in allowing discovery of potential biomarkers from the diseased site. As neurons in the brain are the principal site of rabies virus infection, we selected human frontal cortex as an appropriate site to investigate such proteomic changes. Post-mortem brain tissue has commonly been used for similar proteomic studies in other neurological disorders [16,17].

We sought to carry out a quantitative proteomic analysis of encephalitic and paralytic rabies to identify signature proteins that are differentially regulated. There have been no reported studies to identify differentially regulated proteins in human rabies. Therefore, we carried out iTRAQ-based quantitative proteomics using high resolution mass spectrometry to identify candidate biomarkers in rabies. Using human brain tissues from confirmed cases of encephalitic and paralytic rabies and compared with uninfected normal brain tissues by differential labeling with iTRAQ reagents.

The iTRAQ method has been employed to identify biomarkers in a variety of neurological disorders [18-20]. The multiplexing capability offered by the iTRAQ reagents, which are available as eight different isobaric mass tags, permits simultaneous evaluation of up to eight samples. In the current study, we used the iTRAQ as a method to quantitatively analyze the proteome of brain tissue from cases of encephalitic, paralytic and partially vaccinated rabies, simultaneously comparing with a pool of histologically normal cerebral cortical tissues that serve as a common reference followed by validation of some of the molecules using immunohistochemistry and dot blot assays.

## Results and discussion

**Figure 1:**
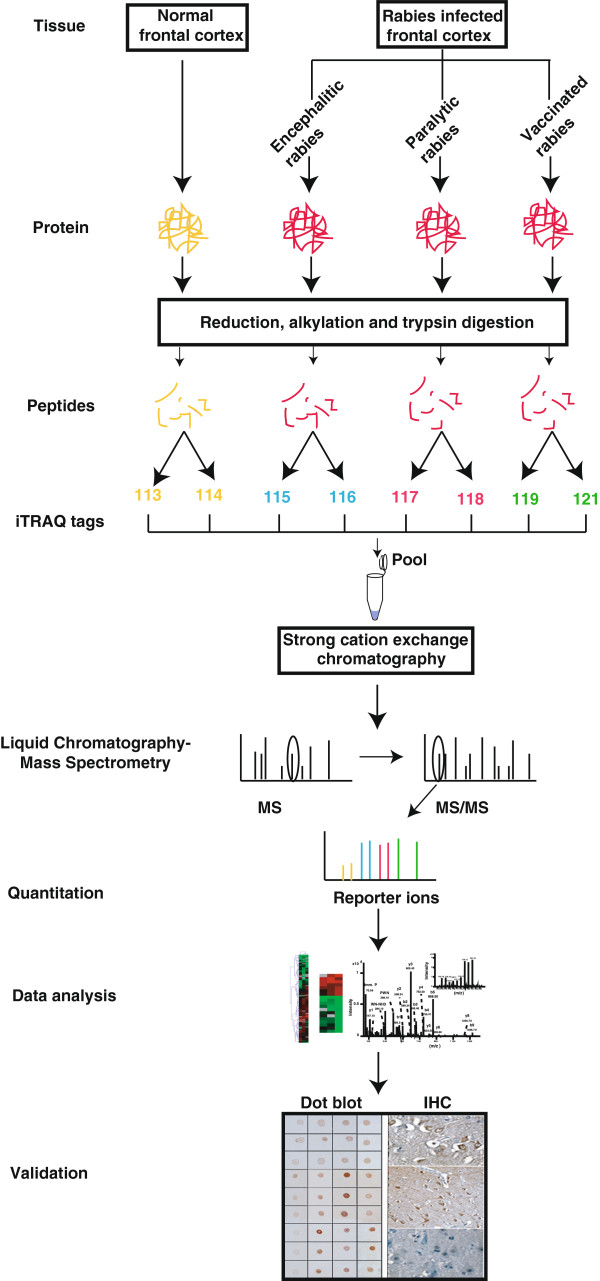
**Various clinical subtypes of rabies tissues were utilized to extract proteins and digested with trypsin.** Peptides from four groups including controls were labeled with iTRAQ reagents followed by LC-MS/MS on a QTOF mass spectrometer. A subset of molecules was validated using dot blot and IHC.

We carried out an iTRAQ based quantitative proteomic profiling of frontal cortex from confirmed cases of rabies viral encephalitis and identified a set of molecules that are differentially regulated in rabies as compared to normal frontal cortex. A set of differentially expressed proteins were identified that may serve as a potential candidate biomarkers to differentiate encephalitic from paralytic forms of rabies. The approach employed for this study is illustrated in Figure 1.

### Quantitative mass spectrometric analysis

The aim of our study was to identify differentially expressed proteins in human brain in cases of encephalitic rabies and paralytic rabies as compared to uninfected control human brain tissues by quantitative proteomics. As shown in the Figure 1, we used two technical replicates, which are lableled by two different iTRAQ tags for each rabies group as well as control. Quantitative proteomic analysis of rabies proteome employing high resolution tandem mass spectrometry coupled to liquid chromatography (LC-MS/MS) led to 59,798 MS/MS spectra from which we identified 402 proteins at 1% FDR. Additional file 1: Table S1 shows differentially expressed proteins and the biological annotations associated with those proteins. Details of combined list of peptides for proteins identified using Spectrum Mill and Mascot are summarized in Additional file 2: Table S2.

We identified 94 proteins as differentially regulated in rabies as compared to normal brain tissues. In cases of encephalitic rabies, 32 proteins were found to be upregulated and 7 proteins were downregulated. Paralytic rabies showed upregulation of 28 proteins and downregulation of 11 proteins while 39 proteins were upregulated in vaccinated rabies and 7 proteins were downregulated.

**Figure 2:**
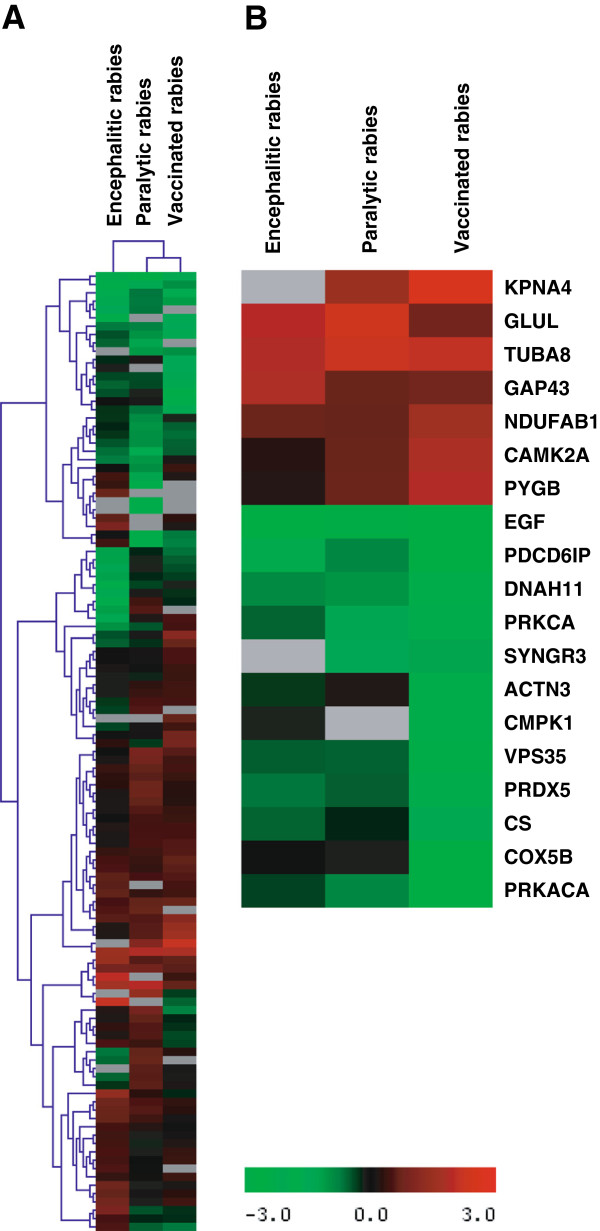
**Hierarchical clustering of proteins identified in rabies. A**. Hierarchical cluster of all proteins. **B**. Hierarchical cluster of differentially regulated proteins in different clinical manifestations of rabies. Protein expression intensities are represented by red and green, for up-regulated and down-regulated fold value, respectively. Black indicates no change in fold value and grey indicates missing value.

To analyze the proteomic data from different clinical conditions of rabies, we employed hierarchical clustering analysis that allows samples which are highly similar in quantitative profiles to be merged in an agglomerative fashion using the average linkage clustering approach. Protein fold-change values as compared to normal brain tissues were employed for this method. We were able to distinguish encephalitic rabies from paralytic rabies by identifying the proteome wide quantitative expression patterns associated with rabies. Hierarchical clustering classified the human rabies samples into two distinct groups based on their fold-changes. As shown in the Figure 2A, paralytic samples clustered in one branch of dendrogram because of the relatedness of their proteome profiles while encephalitic rabies clustered in another branch of the dendrogram indicating differences in expression pattern of proteins. The heatmap pattern also demonstrates that, although most of the proteins showed similar expression pattern, proteomic differences existed between different clinical subgroups of rabies patients. Figure 2B shows a heatmap of upregulated and down regulated protein clusters.

### Functional annotation and classification of differentially regulated proteins

Biological pathway analysis using canonical pathways suggests a significant perturbation in the pathways such as oxidative phosphorylation (7 proteins), tight junction signaling (7 proteins) and calcium signaling (6 proteins) and mitochondrial function (5 proteins). Conti *et al*., have shown that inhibition of replication of rabies virions were correlated with inhibition of oxidative phosphorylation cycle of the host system. This signifies that rabies viral replication is highly energy dependent [21]. Our proteomics study reveals upregulation of seven proteins (ATP5L, ATP5J2, ATP6V1E1, NDUFS3, NDUFB4, NDUFAB1 and UQCR10) involved in oxidative phosphorylation. Rabies employs retrograde axonal transport- that require energy generated through oxidative phosphorylation by the involvement of several mitochondria [22]. Hence, this process could be a suggestive mechanism for the perturbation of pathways such as oxidative phosphorylation, mitochondrial dysfunction and glycolysis. Additional file 3: Table S3 provides the functional pathways associated with rabies. Enrichment analysis of GO terms was performed to examine the functional distribution of the 94 differentially expressed proteins using annotations in Human Protein Reference Database (HPRD, http://www.hprd.org) [23], which follows gene ontology (GO) standards.

Among the differentially expressed proteins, 22% of them are involved in signal transduction, 21% of them are associated with energy pathways and 17% of them are involved in cell growth. Ingenuity pathway analysis also confirmed that proteins associated with encephalitis were enriched including astroglial marker proteins such as GFAP [24,25], oligodendrocyte associated 2′, 3′-cyclic nucleotide 3′ phosphodiesterase (CNP) protein [26,27] and immunity-related GTPase family, M (IRGM1) [28]. According to ingenuity analysis, neurogenesis and guidance of neurites are a few of the important physiological functions which are enriched in our study. Enrichment of these physiological functions is due to the differential expression of proteins such as CTNNB1, Epsin 1 (EPN1) and GAP43, suggesting that physiological functions involving neuronal pathways are active in rabies infection. Axonal morphological changes have also been observed in the rabies infected neurons [3,29].

### Proteins previously known to be associated with rabies

To the best of our knowledge, this is the first quantitative proteomics study of human rabies. Dhingra *et al*. have previously reported the host response in mice to rabies virus using 2D gel analysis with MALDI-TOF mass spectrometry and identified upregulation of proteins involved in ion homeostasis such as H^+^ ATPase subunit a isoform 1 and Na^+^/K^+^ ATPase [30]. Our study also found that the human orthologs of these proteins are upregulated in rabies. Protein Kinase C alpha (PKCA) was found to be down regulated in rabies by our study. PKCA regulates the nucleocytoplasmic distribution of rabies phosphoprotein enabling the rabies virus to respond to the dynamic microenvironment in the host [31]. Our study shows that synapsin 1, a protein localized on the cytoplasmic surface of synaptic vesicles, is downregulated in paralytic rabies. Lewis *et al*. have shown that synapsin 1 co-localizes with rabies virus in neuromuscular junctions [32]. Tsiang and coworkers have shown the inhibitory effects exerted by rabies virions in actin bundling by Synapsin 1. Microtubule-associated protein 2 (MAP-2) was found to be upregulated in encephalitic rabies. Li *et al*. has shown that, pathogenic rabies N2C virus infected neurons exhibited decreased MAP-2 staining in contrast to the attenuated SN-10 rabies virus [33]. Thanomsridetchai *et al*. analyzed the proteome of paralytic and furious form of rabies with different anatomical areas of dog brain which demonstrated a noticeable change in proteome profiles between paralytic and furious rabies [34].

### Novel upregulated proteins identified in rabies

**Figure 3:**
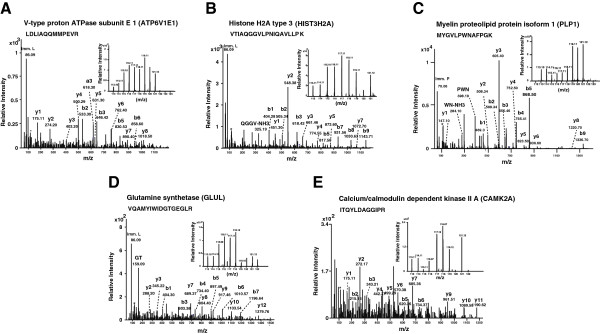
**MS/MS spectra from representative differentially regulated proteins identified in this study.** The inset shows the reporter ions used for quantitation. **A.** Vacuolar H + ATPase E1 isoform b (ATP6V1E1). **B.** Histone H2A type 3 (HIST3H2A). **C.** Proteolipid protein 1 (PLP1) **D.** Glutamate synthetase (GLUL). **E.** Calcium/calmodulin-dependent protein kinase IIA (CAMK2A). Immonium ions for leucine (Imm.L) and proline (Imm.P) are also labeled.

We identified upregulation of 94 proteins in the frontal cerebral cortex of rabies tissues in comparison to the normal human brain tissues. Subsets of the novel proteins, which are differentially expressed, are listed in Table 1. Figure 3 depicts annotated MS/MS spectra of selected proteins, some of which are described below. These proteins include Proteolipid protein 1 (PLP1), which is a transmembrane protein found to be upregulated more than 2-fold in our study. PLP1 is a predominant myelin protein associated with axonal survival. Figure 3C provides annotated spectra for PLP1. Glutamate ammonia ligase (GLUL) is mainly synthesized by astrocytes. GLUL is shown to be associated with schizophrenia [35] moreover, studies in simian immunodeficiency virus infected macaque have also shown dense perineuronal GLUL accumulation thus indicating their role in the host response to viral infection [36]. Moreover, GLUL was also shown neuronal expression in diseases such as Alzheimer’s disease [37]. A representative MS/MS spectrum of GLUL has been provided in Figure 3D. Calcium calmodulin dependent kinase 2 alpha (CAMK2A) is a predominant isoform of Calcium/calmodulin-dependent protein kinase II, a Ca^2+^-activated enzyme abundant in the brain [38]. CAMK2A is 2-fold upregulated in paralytic group compared to other groups in the present study. CAMK2A is also known to be upregulated in peripheral axons after 48 h of inflammation [39]. Increased expression of CAMK2A is also observed in frontal cerebral cortex of cases of schizophrenia [40]. Functionally, CAMK2 is also shown to be expressed in astrocytes and protect the cells from apoptotic stimuli by Fas agonist [41].

**Table 1:** A partial list of proteins differentially expressed in rabies

	**Protein**	**Gene Symbol**	**Features**	**Fold change**		
				**Encephalitic rabies**	**Paralytic rabies**	**Vaccinated rabies**
1.	Contactin 1	*CNTN1*	Over expressed in glioblastoma and localized to the surface of GFAP, normally expressed by neurons and oligodendrocytes.	2.0	1.0	1.7
2.	NADH dehydrogenase (ubiquinone) Fe-S protein 3	*NDUFS3*	NDUFS3 has upregulated in cerebral cortex while decreased in synaptosomal complex as shown by the quantitative proteomic study in animal models of schizophrenia	2.1	1.6	2.6
3.	Syntaxin 1A	*STX1A*	Gene expression studies have shown that high functioning autism patients has increased expression of STX1A compared to normal cases.	2.4	-	3.9
4.	NADH dehydrogenase (ubiquinone) 1	*NDUFAB1*	An acyl carrier protein, which is a part of complex I encoded by mitochondrial DNA. Clinical spectrum of deficiency of complex I include muscle weakness.	2.4	2	3.7
5.	Tumor protein D52	*TPD52*	Calcium/calmodulin-dependent protein kinase mediated phosphorylation of TPD2 showed in rat brain studies.	1.4	2.8	2.4
6.	Calcium/calmodulin-dependent protein kinase IIA	*CAMK2A*	It is a calcium dependent serine/threonine kinase. It is required for hippocampal long-term potentiation (LTP) and spatial learning. It is critical in opioid-induced hyperalgesia	1.6	2.1	4.3
7.	Mitochondrial carrier homolog 2	*MTCH2*	Conditional knock out of MTCH2 has shown decreased Fas-induced apoptosis, possibly by preventing tBID recruitment to mitochondria.	2.7	-	-
8.	Glutamate-ammonia ligase	*GLUL*	Microarray studies has shown that altered expression of GLUL in major depressive disorder.	4.85	2.66	5.48
9.	Aldehyde dehydrogenase 2 family (mitochondrial)	*ALDH2*	Proteomic profiling of human anterior cingulate cortex from patients with schizophrenia has shown differential expression of ALDH2.	1.9	2.2	2.3
10	Growth associated protein 43	*GAP43*	A marker for neuronal regeneration, shown decreased expression in hilus and inner molecular layer of hippocampal formation from patients with schizophrenia.	4.3	2.1	2.6
11		*KPNA4*	KPN4 plays an important role in the nuclear import of viruses such as HIV-1.		3.1	7.3
12	Karyopherin alpha 4	*IPO5*	West Nile Virus enters the nucleus by importin mediated pathway.	2.9	1.9	1.9
13	Importin 5	*PSMA2*	Transcriptomic and proteomic analysis has shown differential expression of PSMA2 in response to enterovirus 71 in rabdomyosarcoma cells.	3.1	2.6	2.5
14	Optic atrophy 1 isoform 2	*OPA1*	Shotgun mass spectrometry study on dorsolateral prefrontal cortex from patients with schizophrenia has shown differential expression of OPA1.	5.1	1.5	1.2
15	Guanine nucleotide binding protein, alpha activating activity polypeptide O	*GNAO1*	GNAO1 functions as a switch in G-protein coupling.	2.2	1.6	2.3

Our study also identified differentially regulated proteins that are associated with retrograde transport (Importin 5) and oxidative stress (Superoxide dismutase 2). Rabies also traverse by retrograde axonal transport. Proteomic analyses of BHK-21 cells infected by rabies virus has shown SOD upregulation suggesting host cells protective mechanism against rabies infection [42]. Apoptosis associated proteins forms a major subset of differentially expressed proteins. These proteins include phosphoprotein enriched in astrocytes 15 (PEA15), Optic atrophy 1 (OPA1), Catenin beta 1 (CTNNB1), Heat shock 70 kDa protein 5 (HSPA5) and Heat shock 70 kDa protein 9 (HSPA9) [43-48].

Moreover, a subset of novel proteins are found to be differentially regulated either in encephalitic rabies or in paralytic rabies, which includes optic atrophy 1 (OPA1), limbic system associated membrane protein (LASMP), hippocalcin-like protein 4 (HPCAL4) and transgelin 3 (TAGLN3). The quantitative differences of the proteome of rabies groups have shown in the Figure 2.

### Novel downregulated proteins identified in rabies

We identified several novel proteins that were downregulated in rabies including adaptor-related protein complex 3, beta 2 subunit (AP3B2), a vesicle coat protein, which is 2.5-fold downregulated in rabies as compared to normal brain. AP3B2 is component of neuron specific AP3 complex. Clinical reports have shown that the downregulation of PDHB presented with neurological dysfunction and high lactate concentration in CSF [49]. Programmed cell death 6 interacting protein (PDCD6IP) also known as Alix is 2-fold downregulated in paralytic as well as vaccinated rabies compared to normal. Functionally, it has been shown that Alix induces apoptosis of neurons by caspase dependent and caspase independent manner besides playing a role in endosomal trafficking [50]. In conjunction, immunohistochemical labeling of Alix has shown an over expression in the cell body of dying neurons from the lateral striatum of Lewis rats that were chronically treated by 3-nitropropionic acid [51]. This data corroborate with previous studies [30] which demonstrates that rabies results in perturbation of host proteins leading to the neuronal dysfunction. Thus, further studies are necessary to elucidate the functional importance of these in host pathogen relations.

### CAMK2A validation by immunohistochemistry

We selected candidate proteins for further validation based on the extent of overexpression, biological importance and available literature that validated their detection in body fluids such as CSF. Differential expression of CAMK2A has been observed in CSF samples in neurological disorders using mass spectrometry based assays such as multiple reaction monitoring (MRM) [52]. Thus, validation of CAMK2A overexpression in the brain tissues could potentially benefit from further testing and evaluation of CAMK2A in the CSF.

**Figure 4:**
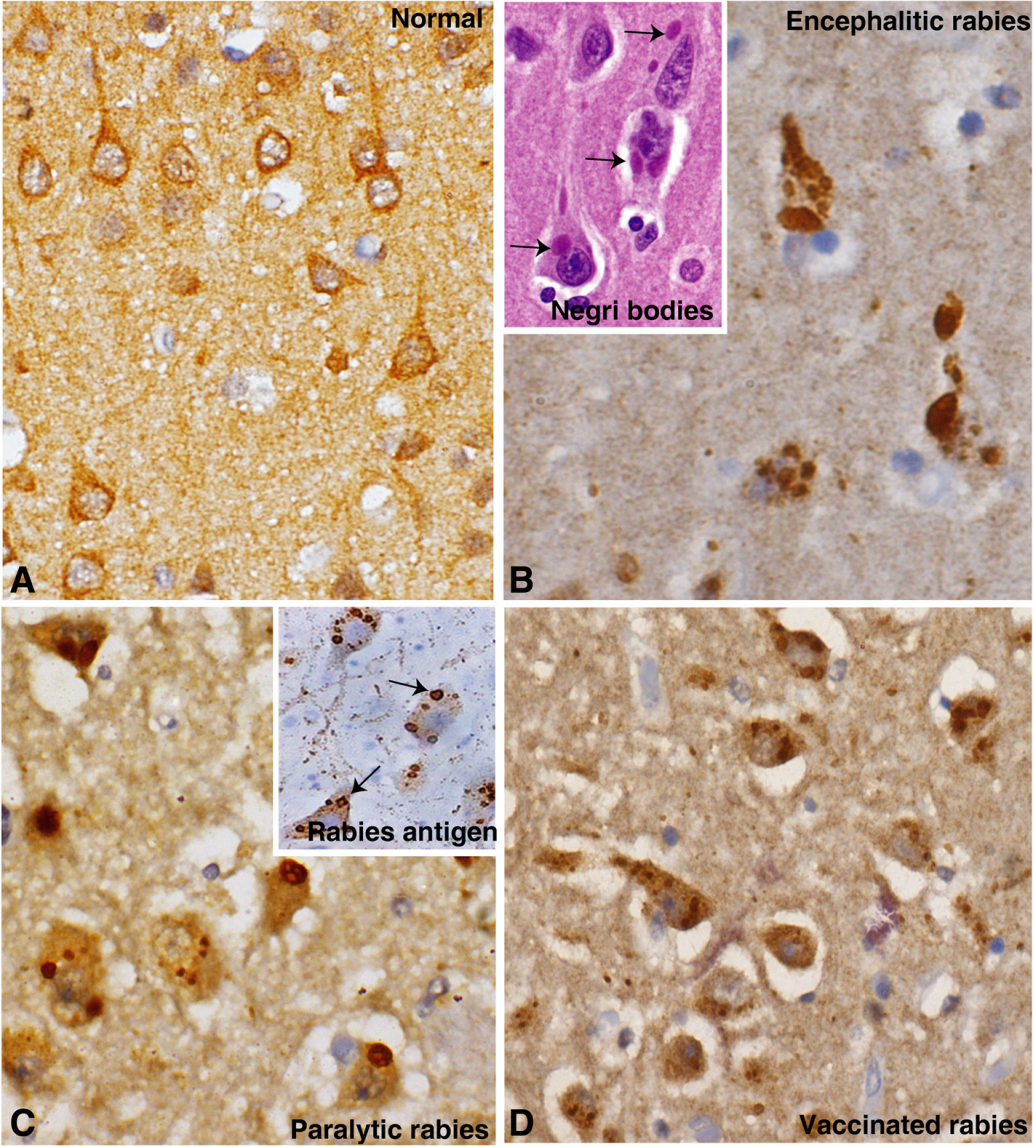
**Immunohistochemical validation for CAMK2A on human cerebral cortex. A:** Normal cortex showing labeling of soma of pyramidal neurons extending along the apical dendrite with diffuse but light synaptic pattern of labeling of the neuropil. **B:** Encephalitic rabies. Hippocampal pyramidal neurons from a case of encephalitic rabies reveal presence of CAMK2A as clumps in the cytoplasm appearing to localize within intracytoplasmic negri bodies (proteomics: 1.6 fold elevation). Inset shows characteristic intracytoplasmic eosinophilic Negri bodies diagnostic of rabies (arrows). **C, D:** Paralytic rabies. In cases of paralytic rabies, labeling with CMAK2A was seen within hippocampal pyramidal neurons, aggregating as coarse granular or clump-like fashion in addition to diffuse labeling of the cytoplasm and neuropil, corresponding to localization of rabies viral antigen (Inset, arrows). This was more intense in vaccinated cases of paralytic rabies **(D)** compared to non vaccinated cases **(C)** (proteomics: 2.08 fold elevation in non vaccinated form, 4.6 fold elevation in vaccinated rabies). [**A**: ImmunoperoxidasexObj.20, **B**: xObj.40, **B**, inset: H&ExObj.40B, **C**: Obj.x20, **C**, inset: Immunoperoxidase to rabies viral antigenxObj.40, **D**: xObj.20].

Evaluation of the histological sections helps to identify the pattern of expression and localization in different brain cells such as neurons and astrocytes apart from being a tool for semi-quantitative analysis of the expression. We selected CAMK2A for validation based on its functional relevance in neuronal signaling and 2 fold over expressed in paralytic rabies. CAMK2A expression in normal cases was seen to label in a diffuse manner (Figure 4A). In contrast, in encephalitic rabies, CAMK2A was seen to show intense labeling. Within the hippocampal pyramidal neurons as well as in the granule neurons of the dentate gyrus, CAMK2A was seen to localize as large aggregates in the cytoplasm (Figure 4B). These aggregates appeared to co-localize within intracytoplasmic Negri bodies seen within these neurons on routine hematoxylin eosin stains (Figure 4B, inset) and as aggregates of rabies viral nucleocapsid on immunohistochemistry. In cases of paralytic rabies, the intensity of labeling with CAMK2A was higher compared to encephalitic rabies. It was seen within hippocampal pyramidal neurons, aggregating as coarse granular or clump-like fashion in addition to diffuse labeling of the cytoplasm and neuropil, corresponding to localization of rabies viral antigen (Figure 4C, D). This was more intense in vaccinated cases of paralytic rabies (Figure 4D) compared to non-vaccinated cases (Figure 4C) though localization to sites of rabies viral accumulation within neurons was similar in both forms.

### CAMK2A and GLUL protein validation by dot blot assays

**Figure 5:**
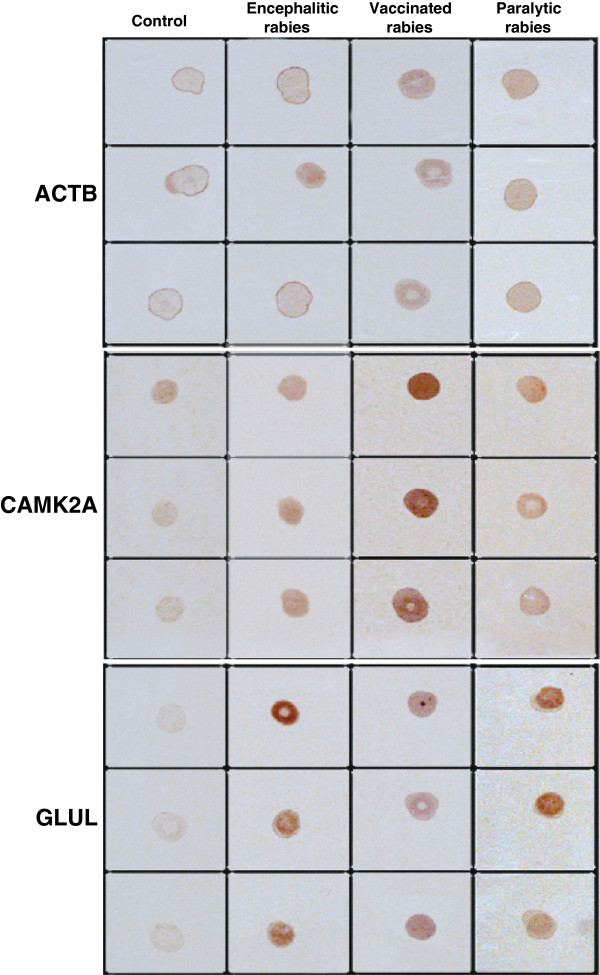
**Dot blot analysis.** Immuno dot blot shows variable expression of CAMK2A and GLUL protein with highest expression of CAMK2A in paralytic vaccinated form of rabies in contrast to encephalitic and non-vaccinated paralytic form. GLUL shows high expression in encephalitic and paralytic (non-vaccinated) with relatively low expression in vaccinated paralytic rabies cases. Blots were run in triplicate as shown in the figure.

Immuno dot blot in Figure 5 shows a variable expression of CAMK2A and GLUL proteins with highest expression of CAMK2A in paralytic vaccinated form of rabies in contrast to encephalitic and non-vaccinated paralytic form. GLUL shows high expression in encephalitic and paralytic (non-vaccinated) with relatively low expression in vaccinated paralytic rabies cases. Detection of GLUL reported in CSF makes it another potential candidate as a diagnostic biomarker [53].

## Conclusions

The diagnosis of rabies in clinical settings is based on symptomatology. We carried out quantitative proteomics approach employing 8-plex iTRAQ and identified 402 proteins associated with human rabies. To the best of our knowledge, this is the first proteomics study of human rabies where we have identified 39 proteins as differentially regulated in encephalitic rabies, 46 proteins in vaccinated paralytic rabies and 39 proteins in paralytic rabies. Many of these proteins are common among the clinical manifestations of rabies. Validation of potential candidate biomarkers like GLUL, CAMK2A and PDCD6IP by employing Immunohistochemistry, Enzyme-Linked Immunosorbent Assay (ELISA) or multiple reaction monitoring (MRM) by mass spectrometry in cerebrospinal fluid or serum will evaluate the effectiveness of these proteins as diagnostic candidate biomarkers in the clinical setting.

## Materials and methods

### Tissue samples

Human brain tissue samples were archived as frozen as well as formalin fixed tissues from autopsy confirmed cases of encephalitic or paralytic rabies and normal brain tissues in the Human Brain Tissue Repository (Human Brain Bank), Department of Neuropathology, National Institute of Mental Health and Neurosciences, Bangalore, India. The study was approved by the Institutional Ethics Review committee of National Institute of Mental Health and Neurosciences, Bangalore with informed consent of close relatives of the deceased. Frozen brain tissue samples were collected from the right frontal cortex from two cases each of encephalitic or paralytic rabies (partially vaccinated or non-vaccinated) and normal controls. All samples were collected within a postmortem interval of 6–10 hours.

The frozen tissues were subjected to pathological confirmation using immunofluorescence tests for rabies nucleoprotein. The control brain tissues considered for the study were confirmed to be negative for rabies or any other infections. Two cases each of encephalitic, paralytic and partially vaccinated rabies were selected for iTRAQ analysis. Encephalitic cases presented in the clinic with predominant limbic symptoms, hydrophobia, aerophobia and phobic or inspiratory spasms. These patients were tested positive by immunofluorescence test for rabies on fresh brain tissues collected postmortem. Patients who presented with paralytic rabies had undergone various neurological investigations including CSF analysis, imaging, and electrophysiological finding to rule out disorders such as Guillain-Barre syndrome. Two of the cases received partial vaccination with Semple’s vaccine with 2 and 6 doses respectively as part of post-exposure prophylaxis. The immunization schedule was incomplete, and no passive immunoglobulin was administered at the site of the bite.

All cases considered for the study were confirmed histopathologically to have characteristic intracytoplasmic Negri bodies within cerebellar Purkinge neurons and pyramidal neurons of the hippocampus, frontal and temporal cortex. Widespread presence of rabies viral antigen within various neuroanatomical regions of the brain, most prominently in limbic areas of hippocampus, temporal cortex, cingulate gyrus, orbitofrontal and insular cortex as well as hypothalamus, brain stem and cerebellum, was detected using antibodies to nucleocapsid antigen of rabies virus by indirect immunoeproxidase technique. The demographic and clinical details are provided in Table 2.

Four micron thick paraffin sections from formalin fixed and paraffin embedded frontal cortex from the same cases that were subjected to proteomic analysis (encephalitic, paralytic and vaccinated and control) along with an additional four paralytic cases (non-vaccinated) were used to validate the expression pattern of some of the candidate biomarkers identified by immunohistochemistry.

### iTRAQ labeling and SCX fractionation

The frontal cortical tissues were homogenized in 0.5% SDS in a Dounce homogenizer followed by sonication. iTRAQ (Applied Biosystems) labeling was carried out according to manufacturer’s instructions. Briefly, 100 μg protein of protein lysate from normal or rabies infected tissue samples of encephalitic, paralytic and vaccinated rabies samples were treated with a reducing agent tris (2-carboxyethyl) phosphine (TCEP) at 60°C for 1 h and alkylated with a cysteine blocking reagent, methyl methanethiosulfonate (MMTS) for 10 min at room temperature. Samples were digested with sequencing grade trypsin (Promega) (1:20) for 16 h at 37°C. Sample digests were further processed by labeling the normal brain-derived peptides with iTRAQ labels of 113 and 114, encephalitic rabies-derived peptides with 115 and 116, paralytic rabies-derived peptides with 117 and 118, and vaccinated rabies-derived peptides with 119 and 121, thus providing technical replicates within a single run. The iTRAQ labeled peptides pooled and fractionated by strong cation exchange chromatography (SCX) on PolySULFOETHYL A column (PolyLC, Columbia, MD) (100 × 2.1 mm, 5 μm particles with 300 Å pores) using an Agilent 1100 series LC system. Sixty SCX fractions were collected using a 0-100% gradient of 350 mM KCl containing 10 mM potassium phosphate buffer (pH 2.85) and 25% acetonitrile for 70 min at a constant flow rate of 0.25 mL/min. The fractions were dried, desalted using C_18_ tips and reconstituted in 10 μl of 2% trifluoroacetic acid before mass spectrometric analysis.

**Table 2:** Clinical and iTRAQ labeling details of the samples employed for quantitative proteomic profiling

	**Sample ID**	**Age/Sex**	**Diagnosis**	**iTRAQ labels**	**Post Exposure Prophylaxis**	**Information of Dog bite**
1.	06/B167	45/M	Normal	113	Nil	Nil
2.	06/B172	35/M	Normal	114	Nil	Nil
3.	A29/02	11/M	Non-vaccinated encephalitic rabies	115	Nil	Nil
4.	A5/08	28/M	Non-vaccinated encephalitic rabies	116	Nil	1 month before death
5.	A8/04	70/M	Non-vaccinated paralytic rabies	117	Nil	2.5 month before death
6.	A61/03	35/M	Non-vaccinated paralytic rabies	118	Nil	2 yrs before death
7	A86/03	30/M	Vaccinated rabies	119	Vaccinated	2 months before death
8	A31/08	28/M	Vaccinated rabies	121	Vaccinated	Right thumb, 20 days

### LC-MS/MS analysis

Desalted iTRAQ labeled peptides were analyzed on a nanoflow LC-MS system containing a chip cube interfaced with Agilent’s 6520 Accurate Mass quadrupole time-of-flight (Q-TOF) mass spectrometer (Agilent Technologies). The chip LC consisted of a 40 nl enrichment column (75 μm × 11 mm) and a 75 μm × 150 mm analytical column made up of Zorbax 300SB C_18_ 5 μm. In reversed phase liquid chromatography (RP-LC), mobile phase A consisted of 0.1% (v/v) formic acid in water, and mobile phase B was 0.1% (v/v) formic acid in 90% acetonitrile. The separation was performed with a linear gradient of B (3% to 40% v/v), at a constant flow rate of 400 nl/min. The ESI source operated in positive mode. Data- dependent acquisition was carried out using MassHunter software with precursor survey scan for 1 second (from 350–1,800 m/z) followed by three MS/MS scans.

### Data analysis

The mass spectrometry data was searched using Spectrum Mill (Agilent Technologies, Version A.03.03) and Mascot (Matrix Science Inc., Version 2.2.0) against human RefSeq Build 35 protein sequence database. Human RefSeq Build 26 (~30,000 protein entries) was used for data search in Spectrum Mill. Searches were carried out with the following search criteria, oxidation of methionine, iTRAQ-8-plex (N-term) and iTRAQ-8plex (K) were selected as fixed modifications and methyl thio-cysteine as a fixed modification. In Mascot as well as Spectrum Mill searches, MS tolerance was set to 100 ppm and MS/MS mass tolerance of 0.1 Da. Only one missed cleavage was allowed. False discovery rate (FDR) was calculated by searching the data against the corresponding reverse database. Peptides with 1% FDR were used to identify proteins. Proteins identified with only one peptide identification from Spectrum Mill or Mascot was further validated by manual inspection of MS/MS spectra.

### Quantitation and statistical analysis

Protein quantitation was carried out using Spectrum Mill and Mascot Distiller (Version 2.3.1.0). Peptides with missing reporter ions were not considered for the calculation of reporter ion ratios. Average intensities of the technical replicates were calculated. Intensity of reporter ions 113 and 114, which corresponds to normal frontal cortex were employed to calculate the fold-changes of proteins against encephalitic rabies (reporter ions 115 and 116), paralytic rabies (reporter ions 117 and 118) and vaccinated rabies (reporter ions 119 and 121) [54]. To improve the visualization of quantitation, we created a hierarchical cluster of proteins identified in rabies infected brain. Proteins, which had a fold-change values of >2-fold were considered as differentially regulated. Hierarchical clustering of differentially regulated proteins was carried out by log base 2 transformations of fold ratios followed by normalization across the samples as provided in MeV. Euclidian distance was used as distance metric and complete linkage employed as linkage method for hierarchical clustering by MeV [55].

### Functional analysis

We employed Ingenuity Pathways Analysis (IPA) software version 7.1 (Ingenuity Systems, Mountain View, CA, USA) for functional analysis. We uploaded proteins which were identified as differentially regulated in rabies as well as their corresponding fold changes. IPA software was employed to compare the data from different clinical manifestations of rabies using single group comparison of IPA, which allows a comparison of different subjects from the same group. Functional annotations and canonical pathways were identified with using a threshold p-value of 0.01 after Fishers exact test. We have annotated localization, biological process and functional class of proteins by mapping gi accession numbers to HPRD accession numbers [56].

### Antibodies

Dot blot was carried out using commercially available antibodies, which were procured from Abcam, Cambridge, MA and directed against GLUL at 1:1000 dilution and CAMK2A at 1:100 dilution were carried out on one case of each group as triplicates. In addition, we also carried out immunohistochemical labeling for CAMK2A at 1:100 dilution from paraffin-embedded sections from the frontal cortex and hippocampus of ten cases of rabies {8 paralytic rabies (2 vaccinated rabies and 6 non-vaccinated rabies), 2 encephalitic rabies} and 4 normal controls.

### Immunohistochemical labeling

The molecules selected for validation were based on the biological relevance and their potential to secretory nature and/or presence of transmembrane domain. We chose CAMK2A in order to validate their potential to act as candidate biomarker. To visualise the immune reaction, Envision kit (DAKO) was used according to the manufacturer’s instructions. Briefly, tissue sections were deparaffinised and antigen retrieval was carried out in citrate buffer (pH 6) by microwaving the tissue sections for 30 minutes. Endogenous peroxidase were quenched by 3% of hydrogen peroxide solution and washed in wash buffer (0.1% Tween in PBS). Sections were incubated overnight with the primary antibodies. After rinsing in wash buffer, the slides were incubated with HRP conjugated appropriate secondary antibody. The signal was developed using Dako chromogen (DAB/Hydrogen peroxidase). The sections were counterstained with Harris’ hematoxylin (blue nuclear stain). The immunohistochemical labeling was assessed by two experienced neuropathologists (AM, SKS) and the findings are summarized in the results section.

### Dot blot assays

For dot blot analyses, 12 μg of protein from each brain tissue lysates were spotted in triplicate onto nitrocellulose membranes, blocked with 2% milk in PBS-T for one hour and then incubated with protein (CAMK2A, GLUL and ACTB) specific primary antibodies for 90 minutes at room temperature. The membranes were washed with PBS-T and incubated with HRP conjugated anti-rabbit secondary antibody – 1:5000 dilution for 90 minutes at room temperature. Secondary antibody treated blots were washed thrice with PBS-T. Protein detection was carried out using DAB based detection system.

### Data availability

Quantitative proteomics data derived from rabies study were submitted to Human Proteinpedia, to make it available for the public [57]. This includes the details of study design, experimental methods, peptides and proteins identified. The entire rabies data could be downloaded from Tranche data repository. This could be performed using Data set (1) "S/BvVTpUE09k0gNOjAP6lbQKxiAzpsd/f92Lu0KfU61PzoxnbGPiS9Hn5EqL3BhAl9HD2 + LReVGAGnoI9QSTFDkaJfAAAAAAABGKwg==” and Data set (2) Iil9DqJYclH4Qvavdq3nvWSfaOWRHI0PgdHpPqH56yFPHT4CqAPBTYFfsYd9TfY1BBoNPRdD9NM2UhFwASv/ClXiv0QAAAAAAAGVaA==” hash.

## Abbreviations

iTRAQ: Isobaric tags for relative and absolute quantification; GLUL: Glutamate ammonia ligase; CAMK2A: Calcium calmodulin dependant kinase 2 alpha; PEP: Post exposure prophylaxis; PVE: Post vaccinal encephalitis; RP-LC: Reverse phase-liquid chromatography; SCX: Strong cation exchange

## Competing interests

The authors declare that they have no competing interests.

## Authors’ contributions

AV designed the study performed the experiment and analyzed and wrote the manuscript. SG performed the experiments and wrote the manuscript. LDS performed the experiments and wrote the manuscript. AM designed the study, analyzed and wrote the manuscript. SR. performed the experiment analyzed and wrote the manuscript. PK Analyzed and edited the manuscript. HP analyzed and edited the manuscript. NS analyzed and edited the manuscript. MS analyzed and edited the manuscript. YR analyzed and edited the manuscript. TKP analyzed and edited the manuscript.SM analyzed and edited the manuscript. HH planned and performed the study. RC designed and wrote the manuscript. PS analyzed and edited the manuscript. AP designed and directed the study and wrote the manuscript. SS designed and directed the study and wrote the manuscript. All authors read and approved the final manuscript.

## Supplementary Material

Additional file 1: Table S1 Differentially expressed proteins identified in rabies. Description of data: List of 94 proteins that are differentially expressed in rabies compared to normal brain tissues. Click here for file

Additional file 2: Table S2 Combined list of peptides for proteins identified using Spectrum Mill and Mascot. Description of data: A list of 402 peptides and their fold changes compared to normal are summarized in Additional file 2: **Table S2**. Click here for file

Additional file 3: Table S3 Functional analysis of the proteins by ingenuity pathway analysis. Description of data: Additional file 3: **Table S3** provides functional annotations of identified proteins as well as p-value with FDR correction. Click here for file
