# Supplementary material for: Quantitative proteomics for identifying biomarkers for Rabies
Source: Clin Proteomics. 2013 Mar 22;10(1):3. doi: 10.1186/1559-0275-10-3 (PMC3660221; doi:10.1186/1559-0275-10-3)
Supplement: Additional file 3: Table S3 — Functional analysis of the proteins by ingenuity pathway analysis. Description of data: Additional file 3: Table S3 provides functional annotations of identified proteins as well as p-value with FDR correction. [file 1559-0275-10-3-S3.doc]

**Supplementary Table 3**

| Functional Annotation | p-value | Molecules | Number of Molecules |
| --- | --- | --- | --- |
| Neurological disorder | 1.78E-05 | ABAT, ACTG1, AK1, ALDH2, AQP4, ATP1B1, ATP5L, ATP6V1E1, CADPS, CAMK2A, CNP, CNTN1, CTNNB1, EEF1G, GAP43, GFAP, GNAO1, GNAQ, GNG2, HSD17B10, HSPA5, HSPA12A, IGSF8, IPO5, MAP2, MAP6, MBP, ME1, MYH9, NDUFAB1, NDUFS3, OPA1, PCP4, PFKM, PLP1, SOD2, STX1A, SYN1, TPD52, TPI1, TUBB4, UBA1 | 42 |
| Skeletal and muscular disorder | 2.57E-03 | ACO1, AK1, ALDH2, AQP4, ATP5L, ATP6V1E1, CADPS, CAMK2A, CNP, CTNNB1, EEF1G, EPB41, GAP43, GFAP, GLUL, GNAO1, GNG2, HNRNPA3, HSPA5, MAP2, MBP, MYH9, NDUFS3, PFKM, RAP1A, SOD2, SYN1, TPD52, TPI1, TUBB4, UBE2L3 | 31 |
| Psychological disorder | 2.98E-03 | ABAT, ALDH2, AQP4, CADPS, CAMK2A, CNP, GAP43, GFAP, GNG2, HSPA5, IPO5, MAP6, ME1, MYH9, NDUFAB1, PCP4, PLP1, SOD2, STX1A, SYN1, TUBB4 | 21 |
| Neurodegenerative disorder | 3.42E-03 | ABAT, CADPS, CNP, EEF1G, GAP43, GFAP, GNG2, HSPA12A, IGSF8, ME1, NDUFAB1, OPA1, PLP1, SOD2, TUBB4 | 15 |
| Alzheimer's disease | 5.24E-03 | ABAT, CADPS, CNP, EEF1G, GAP43, GFAP, GNG2, HSPA12A, IGSF8, ME1, NDUFAB1, OPA1, SOD2, TUBB4 | 14 |
| Huntington's disease | 7.48E-04 | AK1, ALDH2, AQP4, ATP5L, CAMK2A, CTNNB1, GFAP, GNAO1, HSPA5, MAP2, NDUFS3, PFKM, TPD52, TPI1 | 14 |
| Schizophrenia | 3.42E-03 | CNP, GAP43, GFAP, GNG2, IPO5, MAP6, PCP4, PLP1, SOD2, STX1A, TUBB4 | 11 |
| Neurotransmission | 1.06E-03 | CAMK2A, CNP, CTNNB1, EPB41, GNAQ, MBP, PLP1, STX1A, SYN1 | 9 |
| Synaptic transmission | 6.07E-03 | CAMK2A, CNP, CTNNB1, MBP, PLP1, STX1A, SYN1 | 7 |

Functional analysis of the proteins by IPA, wherein the table provides functional annotations of identified proteins as well as p value with FDR correction.
